# Using Layer-by-layer Assembled Clay Composite Junctions to Enhance the Water Dissociation in Bipolar Membranes

**DOI:** 10.1021/acs.langmuir.4c02514

**Published:** 2024-11-13

**Authors:** Nadia Boulif, Menno Houben, Zandrie Borneman, Kitty Nijmeijer

**Affiliations:** Membrane Materials and Processes, Department of Chemical Engineering and Chemistry, Eindhoven University of Technology, P.O. Box 513, Eindhoven 5600 MB, The Netherlands

## Abstract

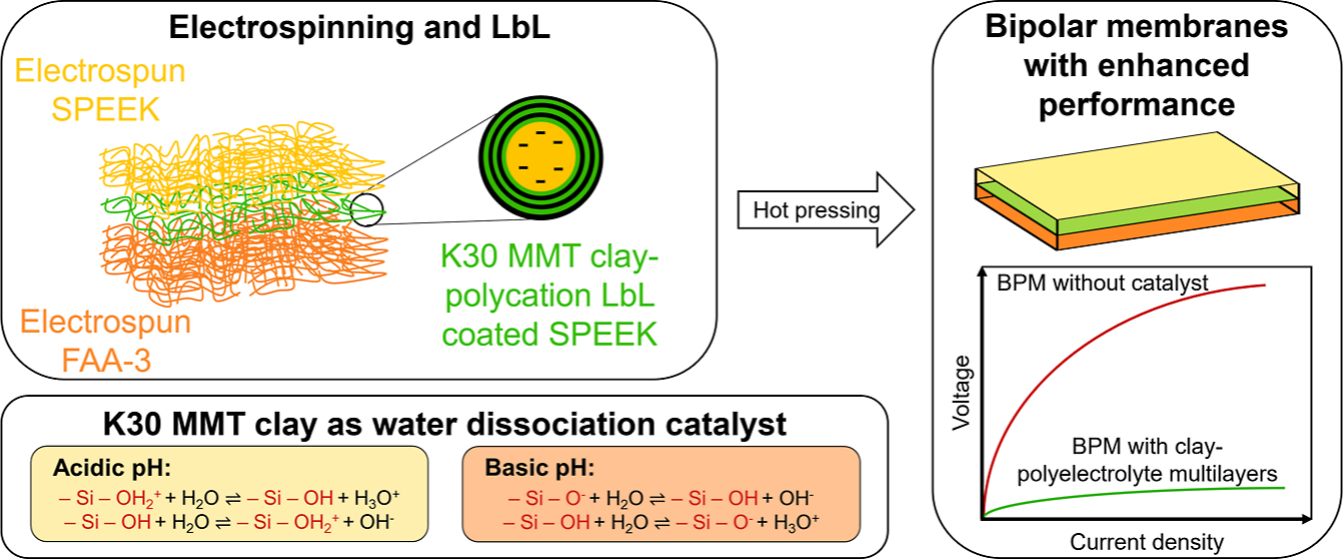

Bipolar membranes (BPMs) with a layer-by-layer (LbL) assembled montmorillonite (K30 MMT) clay-polyelectrolyte (PE) composite junction coated onto a sulfonated poly(ether ether ketone (SPEEK)) electrospun support are prepared, characterized and their water dissociation performance is analyzed. In particular, the focus is on the effect of the presence of the K30 MMT clay as a catalyst for water dissociation, the bilayer number (three, six, and nine), and the PE strength (poly(ethylenimine) (PEI) as a weak PE and poly(diallyl dimethylammonium chloride) (PDADMAC) as a strong PE) on the BPM performance. The BPMs are prepared by electrospinning and hot pressing SPEEK and the Fumion FAA-3 polymer. Adding the composite multilayers in the BPM junction decreases the membrane area resistance in reverse bias from 560 to 21 Ohms cm^2^ for the best-performing modified BPM. The bilayer number has limited influence on the overall membrane resistance, while the PDADMAC BPMs outperform the PEI BPMs due to the higher and more stable PE and clay adsorptions. Electrochemical impedance spectroscopy shows that the depletion layer thickness decreases exponentially with the number of bilayers as the water dissociation reaction becomes less dependent on the junction electric field. Furthermore, the higher Donnan exclusion at the modified junctions improves the BPM permselectivity 3-fold compared to the BPM containing no catalyst. Altogether, these improvements lead to 6.7 times less energy being used in BPM electrodialysis for the production of acid and base when a BPM with composite LBL junction is used compared to a BPM without catalyst. Thus, adding MMT clay composite LbL catalyst to BPM junctions is a promising method to improve the efficiency and reduce the energy consumption of electrochemical processes that rely on BPMs.

## Introduction

Since the first paper reporting their preparation in 1956 by Frilette, bipolar membranes (BPMs) have gained increasing interest for multiple electrochemical applications thanks to their unique ability to dissociate water molecules into protons and hydroxide ions.^1,2^ This property renders BPMs attractive for processes critical for a circular economy, such as resource recovery, electrolysis, CO_2_ reduction, or energy storage.^3−6^

BPMs are traditionally made of a cation exchange layer (CEL) and an anion exchange layer (AEL) laminated together.^2^ This unique combination of both positively and negatively charged layers means that no ion transport can take place through the BPM.^7^ However, if the BPM is placed between two electrodes with the cation exchange layer (CEL) facing the cathode and the anion exchange layer (AEL) facing the anode (or in reverse bias (RB) condition), the applied electric field leads to ion depletion at the BPM junction.^2^ If the electric field generated at the junction is high enough, water molecules at the BPM junction will get split into protons and hydroxide ions according to the second Wien effect (SWE) in order to sustain the ionic current.^8^ Thermodynamically, the minimum voltage required to split water in an electric field is 0.83 V.^9^ The generated ions migrate out of the junction following the direction of the applied electric field: protons and hydroxide ions migrate out of the BPM through the CEL and the AEL, respectively. The generated pH gradient can then be used for several applications such as resource recovery, for example by the acidification of sodium lactate to lactic acid,^3^ or the generation of a pH gradient that enables the use of cheaper electrode catalysts for water electrolysis.^4^

As the water dissociation reaction happens at the BPM junction, it logically follows that the BPM junction conformation and chemistry play a determining role in the BPM performance.^10−12^ A smooth junction obtained by laminating a cation- and an anion-exchange film has the advantage of being a straightforward approach that generates a strong electric field, but has a limited surface area and can easily delaminate due to the geometrically restricted interaction between the AEL and CEL.^2,13^ Recently, more sophisticated approaches for BPM production with enhanced interfacial strength have been investigated. Electrospinning has emerged as a promising technique for the formation of BPMs: the entanglement of the anion and cation exchange fibers increases the surface area and the interaction between the CEL and the AEL, improving both the performance and lifetime of BPMs.^2,11,14,15^ Although the electric field generated in a less planar junction is lower, this can be more than compensated by the addition of a catalyst in the BPM junction: previous studies have highlighted that at high current densities, the catalyst is more determining than the electric field strength for the water dissociation.^16,17^ In the presence of a catalyst, water dissociation happens via a series of (de)protonation reactions, following the so-called “chemical reaction model”. In this model, water dissociation is accelerated by the ability of the catalytic groups to undergo both protonation and deprotonation and/or to promote the formation of “ice-like” water in which the water O–H bonds are weakened by strong hydrogen bonds with the catalyst.^18,19^

Therefore, good water dissociation catalysts are typically materials that contain functional groups that have high (de)protonation reaction rates.^2,18^ Organic acids and bases are good candidates for the water dissociation reaction thanks to their ability to get (de)protonated easily. Examples of such functional groups are carboxylic or phosphoric acid functional groups and amines or pyrrolidines as basic functional groups.^2,14,18,20^ Inorganic Lewis acids such as metal (hydr)oxides are some of the best-reported catalysts thanks to their ability to adsorb and coordinate metastable water, hence facilitating its dissociation.^13^ However, the small size of the metal ions easily results in catalyst leaching out from the BPM junction, leading to poorer BPM performance over time.^20,21^ The incorporation of such catalysts thus necessitates strategies to minimize the metal ion leaching, such as complex formation.^21^

To circumvent metal ion catalyst leakage, 2D nanomaterials that have Lewis acid sites were proposed.^21^ Graphene oxide has already been extensively studied as a very successful catalyst in BPMs but is quite expensive.^9,22−25^ Yet another interesting, abundantly present and cheaper 2D nanomaterial only rarely considered as a catalyst for BPMs is clay. Clays contain silanol groups at their surface that can act as proton donors and acceptors.^26^ Previous papers studied the successful addition of this catalyst in the BPM junction, generating performances similar to those of graphene oxide.^26,27^ However, the investigated incorporation method of the clay was limited to casting a clay layer at the interfacial layer between a cast CEL and AEL, despite convincing evidence in the literature that casting is not the most optimal method for catalyst incorporation in a BPM.^14^ This is due to the SWE’s diminished contribution due to the increased distance between the strong ionic groups of the CEL and AEL, the limited mechanical stability of such interfaces, and their transport limitations.^2,11,15,16^

A more effective method for incorporating organic polymer catalysts in a BPM junction is layer-by-layer (LbL) coating, as it enables the formation of a controllable catalyst layer at the nanoscale.^28,29^ This method can also be translated to composite multilayers containing clays and e.g. polycations, as reported in previous studies for the formation of nacre-mimicking, multiresponsive films (to pH, salt, and temperature), gas barriers or corrosion-resistant coatings.^30,31^ In this process, a polycation layer and a clay layer are alternatively adsorbed on a support. This has been successfully demonstrated on inorganic,^31^ metallic^32^ and polymeric surfaces;^33^ and in combination with different polycations such as polyethyleneimine, poly(*N*-isopropylacrylamide), and poly(diallyldimethylammonium chloride).^31,33,34^ However, to the best of the author’s knowledge, no work has investigated yet the incorporation of clay composite multilayers as a catalyst in a BPM junction.

In this work, we incorporate K30 montmorillonite (MMT) clay in an electrospun BPM via LbL deposition. The CEL and AEL are produced by electrospinning a cation exchange polymer (sulfonated poly(ether ether ketone) or SPEEK) and an anion exchange material (FAA-3). The obtained electrospun mats are then hot pressed together to form a BPM. The K30 MMT is incorporated in the BPM junction via LbL coating on the SPEEK electrospun mat at the interface between the CEL and AEL. The polycation used to form the bilayers is either poly(diallyldimethylammonium chloride) (PDADMAC) as a strong polyelectrolyte or branched poly(ethylenimine) (PEI) as a weak cationic polyelectrolyte. Coating the clays around the electrospun fibers enhances the surface area in which the catalyst can be deposited thanks to the high porosity of the nanofiber mat. Furthermore, the loss of the SWE contribution to water dissociation is limited since there is no thick catalyst layer separating the CEL from the AEL, as the LbL layer thickness is in the order of a few nanometers. Finally, it is expected that the surface area between the electrospun CEL and AEL is coarser and more intermingled than between a cast CEL and AEL, thereby enhancing the mechanical stability of the BPM.

The goal of this work is to study the effect of the employed polycation strength and the bilayer numbers on the properties of the BPM junction which is formed by LbL deposition of clays on an electrospun SPEEK mat.

## Materials and Methods

### Materials

**Figure 1:**
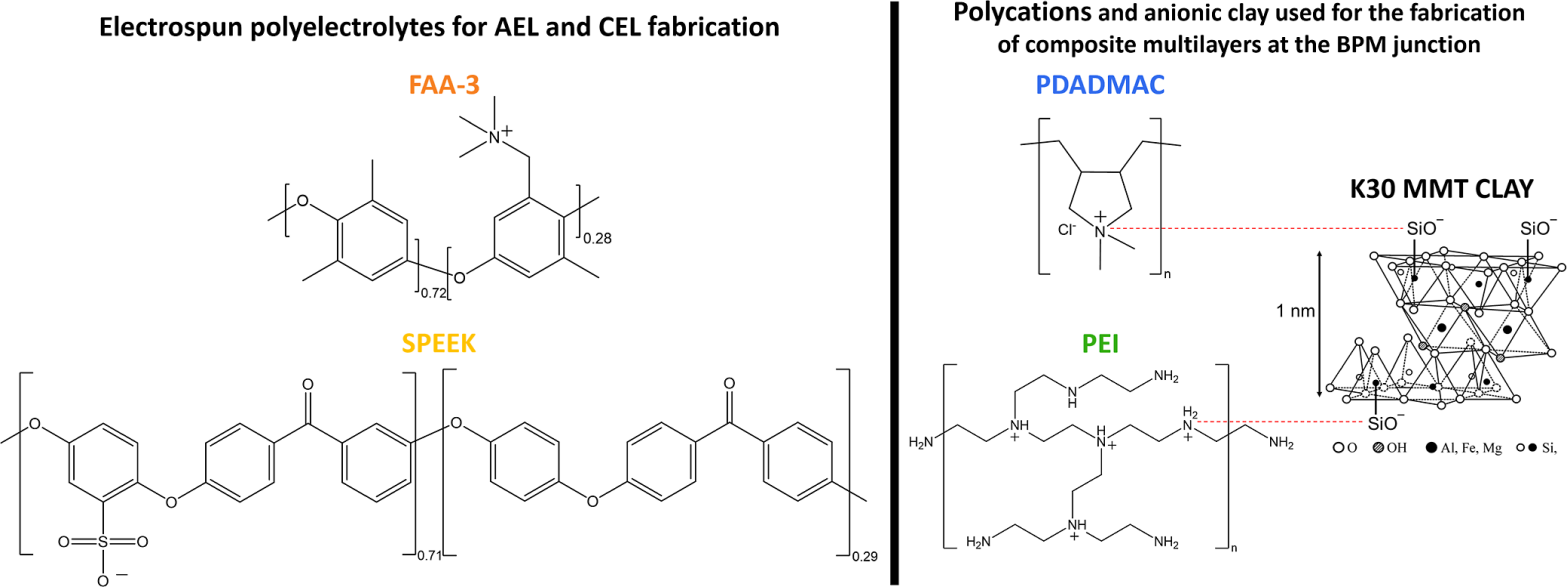
Chemical structure of the used polyelectrolyte and clays. The structure of the K30 MMT clay is adapted from the work of Golubeva and co-workers.^35^ Copyright 2005 by Golubeva. The red lines corresponds to the electrostatic interactions between the positive charges of the polycation and the surface anionic groups of the clay that lead to the formation of layers.

Sulfonated poly(ether ether ketone) (SPEEK, lot number P1910-198, sulfonation degree: 71%), and the anion exchange material (FAA-3, lot number Q0826008, functionalization degree: 28.3%) were purchased from FumaTech GmbH (Germany). *N*,*N*-Dimethylacetamide (DMAC, ReagentPlus, 99%) was purchased from Sigma-Aldrich (Germany). For the LbL, K30 Montmorillonite (K30 MMT, K-catalyst), poly(diallyldimethylammonium chloride) (PDADMAC, *M*_w_: 200–350 kDa, 20 wt % in H_2_O) and branched poly(ethylenimine) (PEI, *M*_w_: 750 kDa, 50 wt % in H_2_O) were purchased from Sigma-Aldrich (Germany). Sodium chloride (Sanal P) was supplied by Nouryon (The Netherlands). For the BPM characterization, 1 M hydrochloric acid (HCl, Supelco from Sigma-Aldrich, Germany), sodium sulfate decahydrate (Na_2_SO_4_·10H_2_O, >99%, Acros Organics, Spain), potassium chloride (KCl, Supelco, Merck, Germany) and sodium hydroxide (NaOH, VWR Chemicals, Czech Republic) were used. Iron(III) chloride hexahydrate (FeCl_3_·6H_2_O) and iron (II) chloride tetrahydrate (FeCl_2_·4H_2_O) were purchased from Sigma-Aldrich (Germany) and were used to prepare the electrolyte. The demineralized water was obtained from an Elga Water Purification System from Veolia (The Netherlands). All chemicals were used as received. The chemical structures of the materials used to produce the BPMs are displayed in Figure 1.

### Experimental Method

#### BPM Membrane Fabrication

##### Electrospinning of the Fibers

The anion (FAA-3) and cation exchange materials (SPEEK) were individually electrospun using a Nanospider NS lab wire electrospinner (Elmarco, Czech Republic). The electrospinning conditions are reported in Table 1.

**Table 1:** Electrospinning Conditions of the Anion Exchange Material (FAA-3) and Cation Exchange Material (SPEEK)

	FAA-3	SPEEK
concentration (wt %)	26	22
solvent	dimethylacetamide dried on molecular sieves	
temperature (°C)	22.0 ± 0.2	
humidity (g/kg)	3.2 ± 0.2 (RH 19%)	4.1 ± 0.2 (RH 25%)
substrate material	siliconized paper 85 g/m^2^ on paper core (Elmarco, Czech Republic)	
carriage orifice diameter (mm)	0.8	
working distance (cm)	24	15
substrate-to-collecting electrode distance (mm)	25	
substrate speed (mm/min)	10	5
carriage speed (mm/s)	100	
applied voltage (kV)	80.0 ± 0.1	

##### LbL Modification of SPEEK Mats

The innermost (closest to the BPM junction) SPEEK mat was modified by LbL to introduce the K30 MMT catalyst. SPEEK mats were cut to the desired size and put in between plastic spacers to prevent the mats from folding. During the whole LbL process, the mats were placed on a KS 4000 I control shaker (IKA-Werke GmbH & Co. KG, Germany) set at 60 rpm and room temperature. The pH of the solutions was adjusted by adding 3 M NaOH when necessary. The mats were first wetted for at least 30 min in a 50 mM NaCl solution with pH 10. They were then put in a 0.01 wt % polycation solution (PEI or PDADAMC, 50 mM NaCl, pH 10) for 15 min, after which they were rinsed in a 50 mM NaCl solution at pH 10 for 15 min. The clay layer was then adsorbed by dipping the mats in a 0.01 wt % K30 MMT clay dispersion (50 mM NaCl, pH 10) for 15 min, after which they were again rinsed in a fresh 50 mM NaCl solution, resulting in the formation of SPEEK fibers coated with one clay/polycation bilayer. The process was repeated as many times as necessary to obtain the desired number of bilayers. In that way, SPEEK mats with three, six, and nine bilayers with either PEI or PDADMAC were obtained. The mats were dried overnight in a vacuum oven (Memmert GmbH + Co. KG, Germany) set at 60 °C before hot-pressing.

##### Hot Pressing of the BPMs

The BPMs were made by superimposing 8 × 8 cm^2^ layers of the desired materials. These were the following: three layers of SPEEK electrospun mats, one LbL-coated SPEEK mat, and four layers of FAA-3 electrospun mats. The electrospun stack was put in between Teflon sheets and subsequently hot-pressed in a LabEcon 300 automated hydraulic laboratory press (Fontijne presses, The Netherlands) for 45 min at 150 °C and under 100 kN, after which they were cooled down to 60 °C under a 100 kN force. The successful densification of the BPM was observed by the formation of transparent BPM samples. The obtained BPMs were named after the used LbL system at the junction (number of bilayers, polycation abbreviation). Additionally, a control membrane that does not contain any catalyst (named “blank”) was produced as reference BPM by hot-pressing four layers of electrospun SPEEK mats and 4 layers of electrospun FAA-3 mats.

#### Characterization Methods

##### Membrane Morphology and Composition (Scanning Electron Spectroscopy (SEM) and Energy Dispersive X-ray Spectroscopy (EDX))

The morphology and composition of the electropsun mats and fibers, and the cryogenically fractured BPM cross-sections were analyzed using a JEOL JSM-IT100 scanning electron microscope (JEOL, The Netherlands). The samples were always sputter-coated with platinum for 60 s at 40 mA using a JEOL JFC-2300 HR (JEOL, The Netherlands) sputter coater to prevent sample charging.

The SEM images were obtained at a working distance of 10 mm using an applied voltage of 10.0 kV and a probe current of 50 nA. The fiber diameter of the electrospun fibers was determined at a magnification of 20,000× by taking an average of 20 randomly chosen fibers. For the EDX analysis, the probe current was adjusted to 69 ± 2 nA until an intensity of roughly 2500 counts per second was registered. The junction thickness was determined graphically from the thickness in which the sulfur and the bromide profiles overlap and change from a high to a low cps count.

##### Optical Fixed-Angle Reflectometry

The formation of clay-composite layers was studied with an optical fixed angle reflectometry setup that was custom-made by Wageningen University & Research (The Netherlands).^36^ Silicon wafers with a silica layer thickness of 66.4 nm were used as a substrate material and were wiped with ethanol, blow-dried, and treated for 5 min with UV–ozone. The polyelectrolyte and rinsing solutions (the same as those used during the LbL modification of the SPEEK mats) were flown in the stagnation point flow cell for 5 min each following the order: rinsing solution, polycation solution, rinsing solution, and clay solution. This cycle was repeated as many times as necessary to obtain the desired number of bilayers. The material adsorption was determined in solution by the change in ratio of the parallel and perpendicular reflected components from a polarized monochromatic light (He/Ne laser 1108P, 632.8 nm, power < 1 mW) that was hitting the substrate at an angle of 70° (Brewster angle).

##### X-ray Photoelectron Spectroscopy (XPS)

The elemental composition of the electrospun SPEEK mat surfaces modified by LbL was determined with X-ray photoelectron spectroscopy (XPS). A K-alpha spectrometer (Thermo Scientific, The Netherlands) was used. A monochromatic (1486.6 eV), 400 μm X-ray spot was generated by an aluminum anode operating at 72 W. The signals were detected with a 180° double-focusing hemispherical analyzer with a 128-channel detector. The survey scans were measured at an energy of 200 eV, and the region scans at 50 eV. Throughout the spectra acquisition, the pressure was kept at 4 × 10^–7^ mbar argon.

##### BPM Permselectivity

The BPM permselectivity was determined by putting a sample between 0.5 M HCl and 0.5 M NaOH in a two-compartment cell with the AEL facing the 0.5 M NaOH compartment as reported in previous works (please refer to Figure S.I.1.1 in the Supporting Information for an optical picture of the setup).^37,38^ One liter of each solution was recirculated as the open cell voltage was monitored for an hour with an Ivium n-stat (IVIUM Technologies BV, The Netherlands). Duplicates were measured for each membrane sample, and two samples were used for each membrane type. The membrane permselectivity was determined according to eq 1
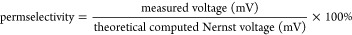
1

The theoretical Nernst voltage for the BPM was determined as the sum of the theoretical voltages determined over the AEL and the CEL, as reported in eq 2
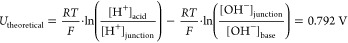
2where *U*_theoretical_ is the theoretically expected OCV for a 100% selective BPM in V, *R* is the gas constant (8.314 J/mol/K), *T* is the solution temperature in K, *F* is Faraday’s constant (96,485 C/mol),  is the proton molarity in the acid compartment (mol/L),  is the proton molarity in the BPM junction (mol/L),  is the hydroxide molarity in the BPM junction (mol/L) and  is the hydroxide concentration in the base compartment (mol/L). The value of 0.792 V is computed for the case in which the acid and base concentrations are of 0.5 M. The proton and hydroxide ion concentration at the junction is assumed to be of 10^–7^ M since the junction is neutral due to the equilibrium between acid and base.

##### Current Density–Voltage Curves

Current density–voltage (*J*–*V*) curves under reverse bias (water dissociation) were measured using a six-compartment cell as reported in previous works (see Supporting Information, Figure S.I.1.2 for an optical image of the setup).^39^ The electrolyte rinsing solution consisted of 0.5 M Na_2_SO_4_, and the buffer and measuring compartment solutions of 0.5 M NaCl. All the solutions were kept in a thermostatic bath set at 25 °C during the measurement. The cation and anion exchange membranes Neosepta CMX-fg and AMX-fg from ASTOM Corp. Ltd., (Japan) were used as separators between the compartments.

The *J*–*V* curve was determined with an IVIUM n-stat (IVIUM technologies BV, The Netherlands) by applying current densities of 0 to 10 mA/cm^2^, and subsequently measuring the voltage drop over the BPM with Haber-Luggin capillaries filled with 2 M NaCl and Ag/AgCl reference electrodes. The obtained *J*–*V* curves were corrected for blank values, i.e., the voltage drop measured without any BPM was subtracted from the voltage drop obtained with the BPM to remove the cable and solution contributions to the resistance. Each membrane sample was measured twice and two membrane samples were measured for each BPM type. The onset potential of the BPM was determined as the voltage at which the tangent of the first three points (in the co-ion transport region) and the last three points (in the water dissociation regime) intercept each other.

The same setup was used for the *J*–*V* curve determination at higher current densities, except that the membrane active area was reduced from 17.3 to 0.79 cm^2^ to prevent excessive O_2_ and H_2_ formation at the electrodes. The current density was then varied from 0 to 100 mA/cm^2^ in steps of 10 mA/cm^2^.

##### Electrochemical Impedance Spectroscopy

Further insight into the contribution of the ion transport, the water disproportionation reaction, and junction capacitance was gained using electrochemical impedance spectroscopy (EIS). The BPM EIS spectra were measured using the six-compartment setup described in the previous section.

An alternating current of 173.500 mA (corresponding to a current density of 10 mA/cm^2^) with an amplitude of 50% (±5 mA/cm^2^) was applied with frequencies ranging from 100 kHz to 10 mHz with 8 points per decade and 6 measures per frequency using a SP-300 potentiostat (BioLogic, France) connected to a VMP3B-20 BioLogic booster (BioLogic, France). The resulting EIS data were analyzed with the EC-Lab software (V11.50) using the *Z*-fit function. The BPM ohmic area resistance, water dissociation area resistance and junction capacitance were extracted by fitting the Nyquist plot to an equivalent circuit model displayed in Figure 7.

##### BPM Efficiency under Reverse Bias

The efficiency and energy consumption for the acid and base generation via BPM electrodialysis were experimentally determined for a selection of the lab-made BPMs. The setup used for this purpose was as reported in a previous work.^40^

200 mL of an electrolyte containing 0.25 M FeCl_2_, 0.25 M FeCl_3_, and 0.1 M HCl was circulated at 150 mL/min near the platinized titanium mesh electrodes. 200 mL of 0.5 M NaCl was circulated at a flow velocity of 1.25 cm/s in each of the acid, base, and salt compartments (3.2 mm thick). A current density of 5 mA/cm^2^ (in reverse bias for the BPM) was applied to the membrane for 1 h. The pH of the solutions was measured with a pH7110 inoLab pH-meter (Xylem Water Solutions Nederland B.V., The Netherlands) before and after the experiment. The pH of the basic samples was measured after diluting them 10 times to lower the response of the pH probe to the sodium ions and improve the measurement accuracy. The current efficiency of the acid and base production was computed using the following formula
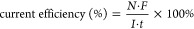
3where *N* is the number of moles of produced protons or hydroxide ions produced (moles) determined from the difference in pH values before and after the experiment, *F* is Faraday’s constant (96,485 C/mol), *I* is the applied current (A) and *t* is the experiment time (3600 s).

The energy consumption for the production of 1 kg HCl was computed using the following equation
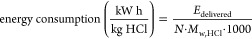
4Where *E*_delivered_ is the energy delivered to the stack extracted from the EC-Lab software (V11.50) (kW h), *N* is the amount of moles of protons produced determined from the change in in pH (moles) and *M*_w,HCl_ is the molecular weight of hydrochloric acid (36.46 g/mol). The factor 1000 is used to convert the g to kg.

The experiments were done in duplo with a different BPM sample for each electrodialysis experiment.

## Results and Discussion

### LbL Quantification

**Figure 2:**
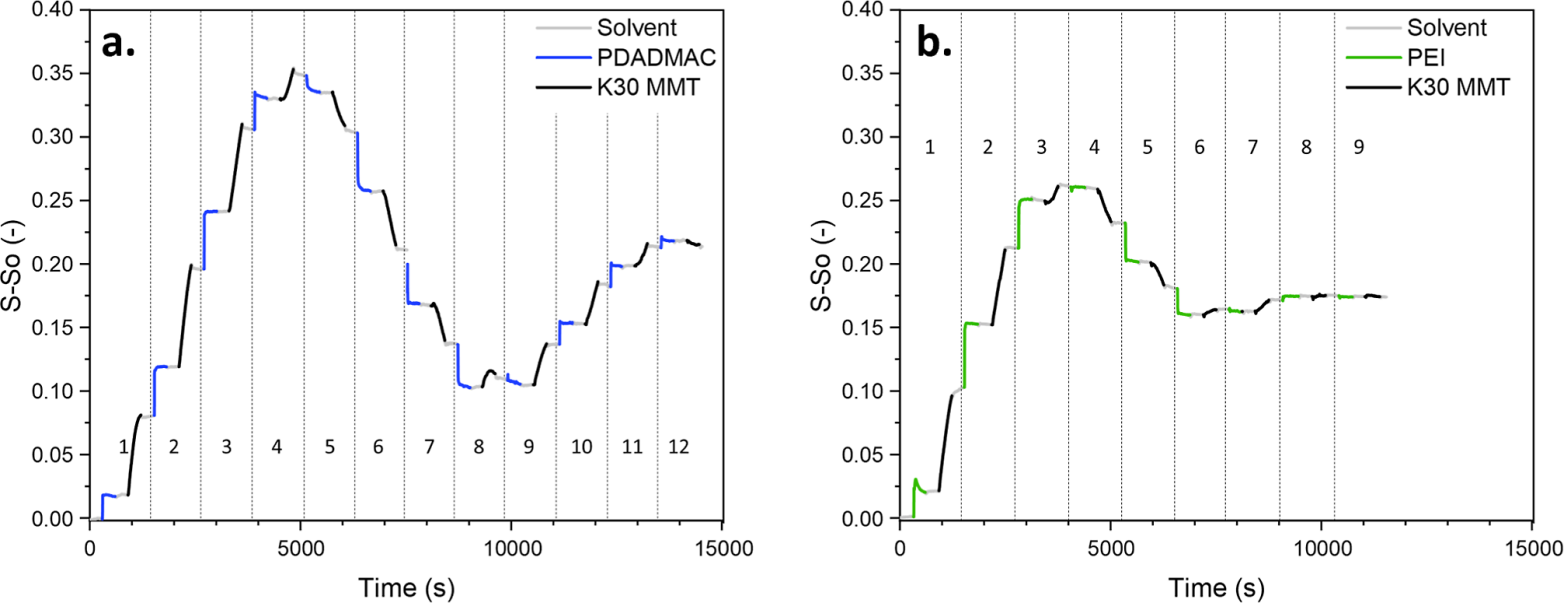
Reflectometry results of the K30 MMT clay composite bilayer formation with (a) PDADMAC and (b) PEI. In all cases, the polycation or clay concentration is 0.01 wt %, 50 mM NaCl, and a pH of 10. The rinsing solution had the same composition as the polyelectrolyte solution (50 mM NaCl and pH 10). The numbers in the graph correspond to the bilayer number.

The build-up of K30 MMT-polycation bilayers using LbL was checked with optical reflectometry and XPS. The reflectometry results are displayed in Figure 2.

Figure 2 shows that while the PDADMAC and PEI adsorption onto the silicon wafer follows the expected stepwise reflectometric signal (which suggests fast adsorption of a stable layer), the clay layers display a steady S–S_0_ increase with time. Yang et al. hypothesized that the dependence of clay platelet adsorption with time is caused by the small size of the clay platelets.^41^ With short coating times, the small clay platelets fill first the valleys of the polymer structure, while the longer deposition times lead to a more uniform and smooth surface that is fully covered by clays. This suggests that the clay platelets are smaller than the defects and valleys of the polymer layer and are unable to bridge them, hence the slower kinetics for the clay layer than for the polymer layer adsorption.

Furthermore, it is interesting to note that the S–S_0_ signal starts decreasing with increasing amount of layers after 4 PDADMAC—K30 MMT bilayers and 3.5 PEI—K30 MMT bilayers, which suggests either material desorption or a decrease in the refractive index of the bilayers. Desorption is less plausible since this contradicts the findings of many studies on the formation of clay and polycation bilayers that measure a steady increase in material adsorption with an increasing number of bilayers using ellipsometry, atomic force microscopy, quartz microbalance, or reflectometry in the dry state.^31,34,41^ These are all techniques that measure the film thickness in the dry state, and this hints at the fact that the decrease in signal observed in this study is most likely due to the swelling of the bilayers in the wet state. This is further confirmed by the fact that the signal decrease follows the same trend as the signal increase: stepwise in the case of the polycation and logarithmic in the case of the clays.

The S–S_0_ signal oscillation observed in the case of PDADMAC-K30 MMT layers is similar to that observed by Buron et al. for poly(trimethylammonium ethyl methacrylate chloride) and poly(acrylic acid) multilayers. They attributed this behavior to the changing refractive index of the layers during the build-up and the associated different diffusion of the laser beam as the multilayers grow due to topological and refractive inhomogeneities in the multilayers.^42^ The results also show that the S–S_0_ signal stabilizes for the PEI-K30 MMT system after 6 BL, which might indicate that there is no more bilayer formation after 6 BLs of PEI-K30 MMT. Therefore, it is difficult to conclude with certainty whether the S–S_0_ decrease is due to desorption or due to changes in optical properties that take place during the layer build-up.

**Figure 3:**
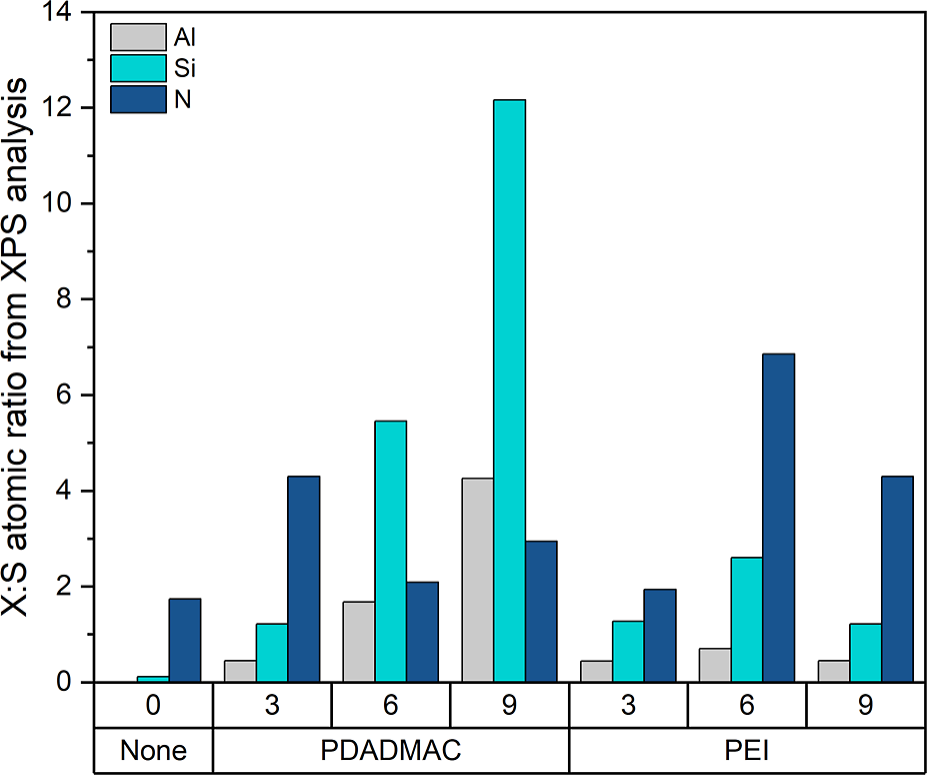
Ratio of Al, Si, and N to S based on the atomic percentage composition XPS results for the LbL coated electrospun SPEEK mats with different amounts of BL. Al and Si are used to track the presence of K30 MMT and N the presence of the polycation. The number on the *x*-axis corresponds to the number of bilayers and the name of the used polycation results.

Next, X-ray photoelectron spectroscopy (XPS) was used to determine the layer build-up on the coated SPEEK mats and the results are displayed in Figure 3 (please refer to Figure S.I.2.1 for the full XPS spectra and S.I.2.2 for the zoom into the S, N, Si and Al signals in the Supporting Information). Figure 3 displays the Al, Si, and N to S ratio for all the LbL-modified electropsun SPEEK mats. The former two elements (Al and Si) are used to track the build-up of the clay layers, while the N-signal is used to track the build-up of the polycation layers.

For the PDADMAC samples, the clay layer build-up follows an exponential trend as seen from the Si/S and Al/S ratios. This exponential layer formation is faster than the reported linear layer formation for poly(vinyl alcohol) and MMT, poly(allylamine) and MMT, poly(*N*-isopropylacrylamide) and MMT or branched PEI and MMT that have been probed with other techniques.^31,41,43,44^ It should be noted that the XPS penetration depth (<10 nm) is quite small relative to the thickness of the nanometer-thick bilayers.^45^ Therefore, as more bilayers are deposited onto the SPEEK, the thickness of the coating increases, which leads to less SPEEK (so sulfur) being probed, hence artificially increasing the Al, Si, or N to sulfur ratio. Nonetheless, that can also be interpreted as yet another indication that the thickness of the LbL film grows with the addition of more bilayers. Regarding the N/S ratio, this shows a decrease from 3 to 6 BL PDADMAC, which is unexpected based on the Al/S and Si/S trends. However, some nitrogen was also probed in the uncoated SPEEK sample, which suggests that the N signal is sensitive to contaminations, for example, residual DMAC from the electrospinning, and is therefore not reliable to track the BL build-up.

For the PEI samples, the Al/S, Si/S, and N/S ratios are multiplied by 1.6, 2.04, and 3.6, respectively when the BL number increases from 3 to 6. However, a decrease in all the ratios is observed from 6 to 9 BL PEI, suggesting material desorption as more BLs are formed. This contradicts the findings from Yang et al. who studied the influence of the deposition time on the LbL growth of branched PEI and MMT and found that increasing the number of bilayers leads to an increase in film thickness.^41^ The difference between this work and their deposition conditions is the lower polyelectrolyte concentration and the addition of 50 mM NaCl in our case. This leads to the adsorption of less material and more open structures in this work, which renders the LbL build-up difficult at higher BL numbers as the interaction with the SPEEK substrate should not any more affect LbL build-up with thicker films.^46^ Furthermore, the low charge densities of PEI (40% at pH 10) and the MMT (due to the acid treatment by the manufacturer) lead to the formation of less stable layers since there are fewer ionic linkages between the layers.^36,41,47^

Based on the above analyses, it can be concluded that the formation of clay-polycation bilayers on electrospun SPEEK is obtained with a strong polyelectrolyte such as PDADMAC but is limited to a few bilayers when using a weak polyelectrolyte with a low ionization degree such as PEI under the conditions used in this work.

### BPM Membrane Morphology

**Figure 4:**
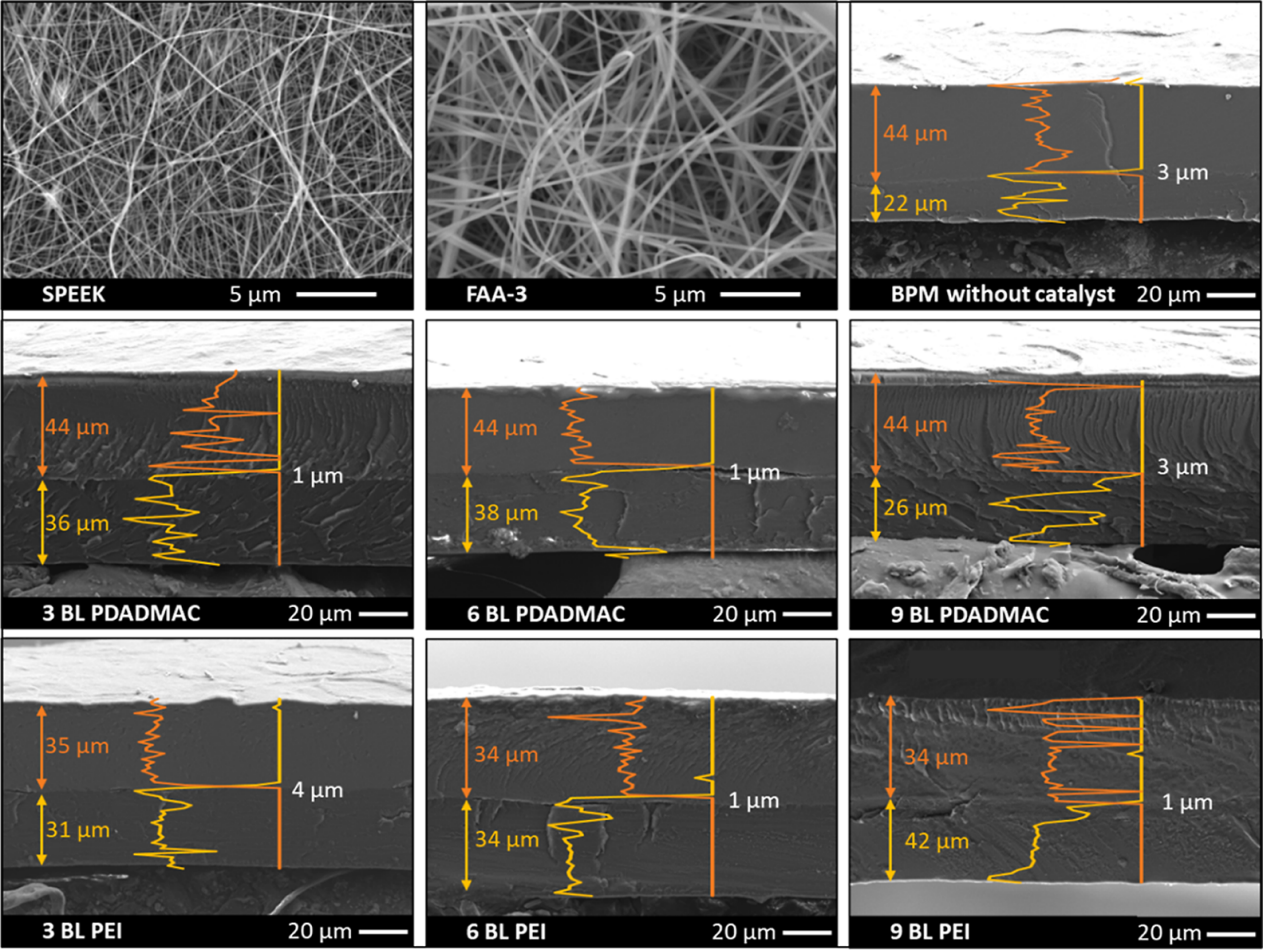
SEM images of the electropsun CEL (SPEEK) and AEL (FAA-3) (top view) and the BPM cross-sections, as indicated in the labels at the bottom. The orange and yellow profiles correspond to the normalized intensity of bromide (AEL) and sodium (CEL), respectively, as detected with EDX analysis. The orange number (top) corresponds to the thickness of the AEL and the yellow number (bottom) to the CEL thickness in μm, as extracted from the EDS profiles. The white number corresponds to the junction thickness extracted from the thickness of the region ins which the sodium and bromide profiles overlap.

After the successful LbL formation of composite multilayer coatings, the BPMs were produced. Figure 4 shows the morphology of the fibers obtained by electrospinning and the BPM cross-sections with the normalized intensities of the counter-ions of each layer. The counter-ions, namely sodium and bromide were used to identify the CEL and the AEL, respectively.

Electrospinning leads to the formation of defect-free fibers with no beads for both SPEEK and FAA-3. The fiber diameter of SPEEK (115 ± 37 nm) is smaller than that of FAA-3 (277 ± 43 nm) due to the lower polymer concentration used in the former case.

Figure 4 shows that the lab-made BPMs have roughly the same thickness (on average 73 ± 7 μm) with similar AEL and CEL thickness as well. There is no difference in thickness between the samples since the bilayers thickness is in the order of a few nanometers, which is not visible in the micrometer scale. The obtained BPMs also have a dense structure where the pores of the electrospun mats are no longer visible, indicating successful hot pressing. The junction thickness is determined by the thickness of the region in which the sodium and the bromide profiles overlap (and thus, the thickness in which the CEL and the AEL are intermixed) and is a maximum of 4 μm. It should be noted that some BPMs visually seem to display some defects at the junction where the CEL and AEL no longer adhere to each other. It is unsure whether the defects are also present in the as-made BPM or are an effect of drying the SEM samples. This can be due to the non-3D entangled junction in this work, as well the coating of the SPEEK fibers with the inorganic clays, which do not have a *T*_g_ and can therefore not fuse with the FAA-3 polymer in the AEL.

### Current Density–Voltage Curves

**Figure 5:**
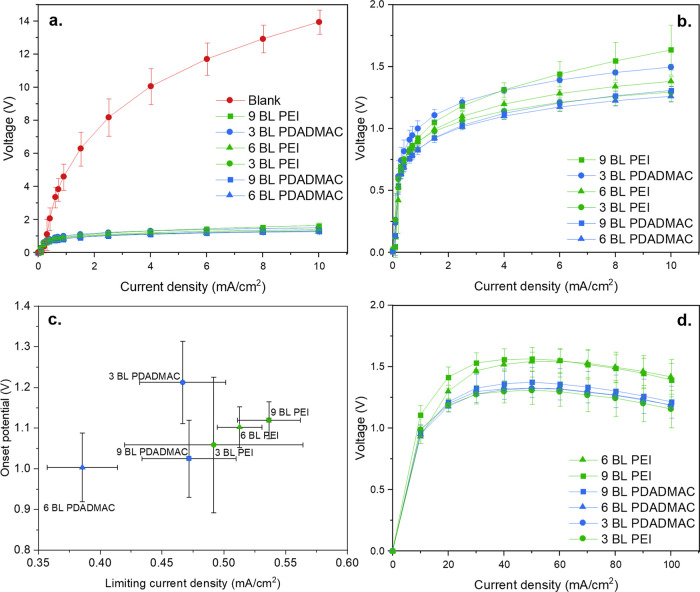
(a) Current density–voltage curves of the lab-made membranes for current densities of 0 to 10 mA/cm^2^. The legend is organized in order of descending potential at 10 mA/cm^2^. (b) Zoom into the lower potential region of 5a. (c) Onset potential vs limiting current density of the BPMs with a polycation-MMT composite multilayer junction, extracted from 5b. (d) Current density–voltage curves of the lab-membranes for current densities of 0 to 100 mA/cm^2^.

Figure 5 shows the current density–voltage curves for the different BPM samples. The blank BPM refers to a control BPM in which no BL is coated and only contains electrospun SPEEK and FAA-3 mats hot-pressed together.

Figure 5a illustrates that the addition of composite LbL coatings drastically lowers the overpotential for water dissociation at 10 mA/cm^2^ from ≈14 V for a BPM without a composite multilayer junction to less than 2 V for the BPMs with polycation-clay bilayers at their junction. This is thanks to the addition of the K30 MMT clay that acts as a water dissociation catalyst through its surface silanol groups that can get (de)protonated.^26^ The performance of the BPMs with polycation-clay multilayers reported in this work have a similar overpotential to a commercial Ralex BPM at 8 to 10 mA/cm^2^, but still have a higher overpotential than the commercial FBM Fumasep BPM.^40^ On the other hand, the BPMs in this work outperform previously reported BPMs with PEDOT:PSS/PEI multilayers by at least 1.5 V at 100 mA/cm^2^.^28^ This is attributed to the higher catalytical activity of the K30 MMT clay compared to PDEDOT:PSS, as well as coating an electrospun SPEEK mat instead of a flat surface with multilayers, which increases the surface area on which the catalyst can be adsorbed.

Figure 5b provides a zoom into the lower potential region of Figure 5a and shows that there is a limited significant difference between the different polymer types and the number of bilayers. This suggests that only the surface of the coated fibers (or the last half bilayer, which consists of MMT clay) is relevant for the reaction catalysis. This hypothesis is further confirmed by the fact that there is no significant difference between the PEI and the PDADMAC samples, although the XPS results suggest that there is more clay adsorption in the case of PDADMAC. In addition, the lack of significant difference between the PEI and PDADMAC samples shows that the polymer layers do not contribute to the catalysis, since amine groups of PEI have a catalytical activity of 0.1 s^–1^, whereas the quaternary ammonium groups of PDADMAC have no catalytical activity toward the water dissociation reaction.^2^ Still, there is no significant difference between the BPMs made from the different polycations.

The limiting current density and the onset potential for water dissociation can be extracted from the intersection of the tangents at 0 and 10 mA/cm^2^ and are plotted in Figure 5c. In the case of PDADMAC, the limiting current density is the smallest for the 6 BL PDADMAC, which suggests that this membrane has the highest permselectivity. There is a sharp drop in the onset potential from 1.2 to 1.0 V between 3 and 6 BL PDADMAC, while the onset potential remains similar for the 6 and 9 BL PDADMAC BPMs. This indicates that there is a threshold number of bilayers above which adding catalysts does not improve the water dissociation further. This finding is similar to that of Eswaraswamy and co-workers who showed that BPMs with a cast MMT interfacial layer did not have improved performance at a clay loading above 1.0 mg/cm^2^.^26^ Similarly, in this work, coating more than 6 BLs PDADMAC does not benefit the BPM water dissociation.

Interestingly, the trends are slightly different for the PEI BPMs as the 3 BL PEI membrane displays the smallest onset potential (1.1 V) and limiting current density (0.49 mA/cm^2^), while the 6 and 9 bilayers PEI BPMs have higher limiting current densities (0.51 and 0.54 mA/cm^2^, respectively). Therefore, it appears that applying more bilayers of PEI and K30 MMT negatively affects the activation energy of the water dissociation reaction. This is similar to the findings of Adbu et al., who also found worse performance after coating the BPM junction with more than 2 bilayers of PEDOT:PSS and PEI.^28^ Care should be taken that these differences are not very significant in the case of PEI.

Several factors contribute to a worsened performance with increasing BL number for PEI, whereas the PDADMAC bilayers display an improvement from 3 to 6 BL, followed by a stagnation in the BPM performance as the BL number increases from 6 to 9 BL. First, as seen in the XPS results (Figure 3), the weak PEI polyelectrolyte leads to less MMT clay being adsorbed on the SPEEK fibers, which leads to a lower catalyst loading. Furthermore, it has been reported that layers formed by weak polyelectrolytes are thicker than those formed with strong polyelectrolyte.^46^ A larger separation between the CEL and AEL strong ionic groups in the case of PEI leads to a lower contribution of SWE to the water dissociation reaction, as previous research has indicated 0.1 nm being an effective distance for the SWE, while the LbL thickness is in the order of nanometers.^48^ In addition, the loss of the SWE contribution is less in the case of PDADMAC as the polycation contains strong quaternary ammonium ionic head groups, whereas PEI only contains weak basic groups that do not contribute to the electric field enhancement at the junction. Therefore, the SWE is more reduced with more PEI bilayers but not with more PDADMAC bilayers.

The water dissociation area resistance is further probed at higher current densities as displayed in Figure 5d. Unexpectedly, the polarization curves display a negative ohmic resistance from 50 mA/cm^2^ onward. This is attributed to the fact that the Na^+^ and Cl^–^ ions present in the CEL and AEL as counter-ions are replaced by the more mobile H^+^ and OH^–^ as the current density (and thus, water dissociation rate) increases. There is no significant difference between the BPMs at higher current densities, besides the 6 and 9 BL PEI BPMs that perform worse than the other BPMs. This is in line with the explanations provided earlier: a weaker polyelectrolyte leads to less catalyst adsorption onto the SPEEK fiber and produces thicker layers without strong ionic groups that lead to less contribution of the SWE to the water dissociation reaction, hence worse BPM performance.

### Electrochemical Impedance Spectroscopy Results

**Figure 6:**
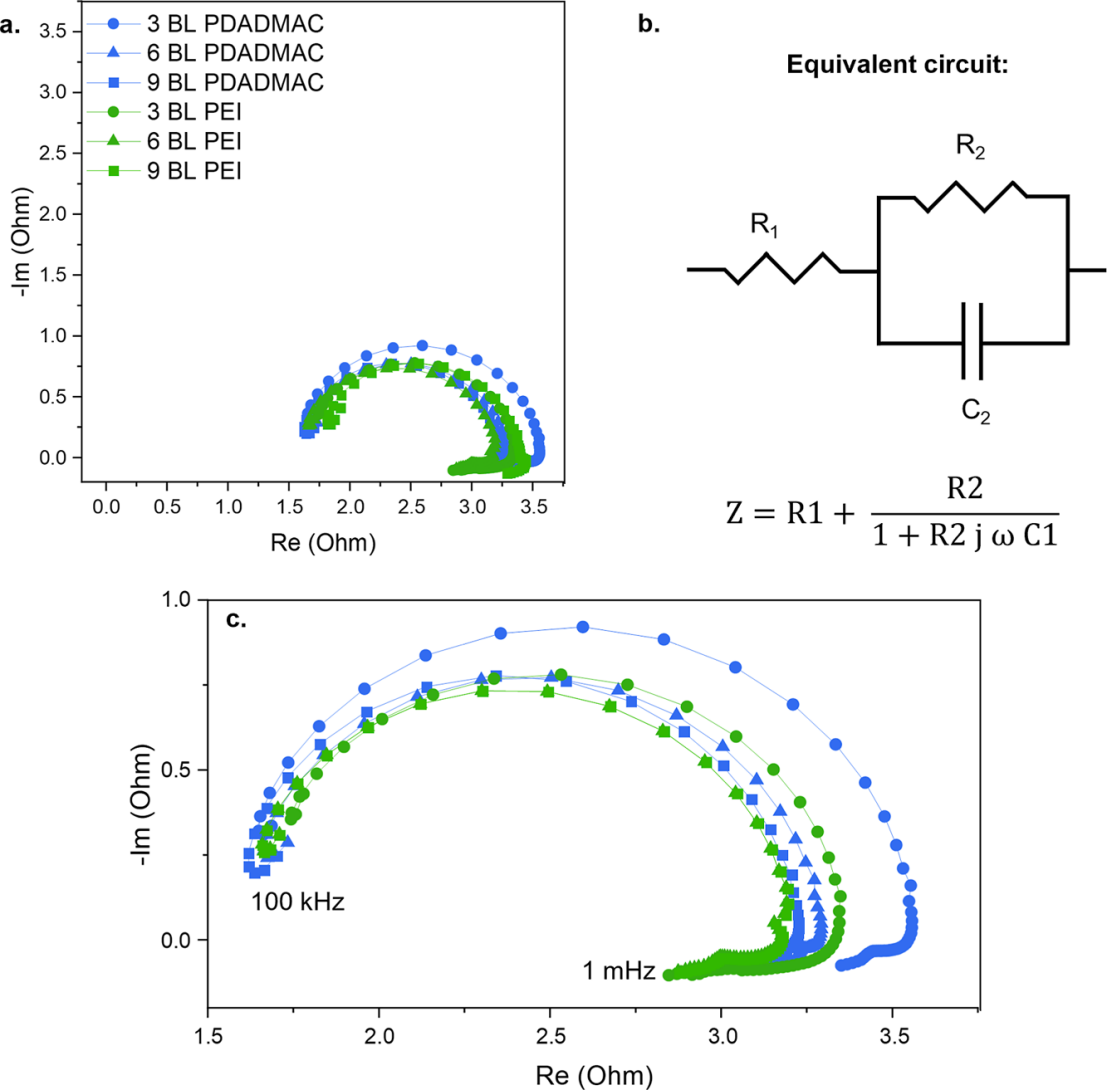
EIS results of the LbL modified BPMs: (a) Nyquist plot of the BPMs with composite bilayers using an orthonormal scale. (b) Equivalent circuit used to fit the data and corresponding equation. *Z* is the impedance, *R*_1_ is the ohmic resistance, *R*_2_ is the water dissociation resistance, *j* is the imaginary component of the impedance, ω is the angular frequency and *C*_2_ is the BPM junction capacitance. (c) Zoom of the relevant region of the Nyquist plot shown in 6a, nonorthonormal scale used.

While the previously discussed *J*–*V* curves provide useful information on the application-focused BPM performance, they provide little insight into which phenomenon contributes the most to the membrane resistance. Electrochemical impedance spectroscopy (EIS) can be used to decouple the contribution of the different transport phenomena to the BPM resistance. The results of the EIS measurements are displayed in Figure 6.

Figure 6a shows that almost perfect semicircles are obtained, which implies that the most suitable equivalent circuit to describe a BPM is an ohmic resistor followed by a resistor and capacitor in parallel as displayed in Figure 6b.^7^ The fit was performed for the obtained data points between 42 kHz and 1 Hz. At frequencies higher than 42 kHz, a small inductance loop is present that is mostly due to cable interferences. Therefore, these points were not included in the fit as this behavior has little to do with the actual performance of the BPM itself. Oddly, an inductance behavior was also observed at frequencies lower than 1 Hz. Pivac and Barbir attributed this behavior in proton exchange membrane fuel cells to side reactions with intermediate species and water transport.^49^ As there is most likely no side reaction happening at the BPM junction, water transport is most likely the cause of this effect. Due to the large amplitude used during the EIS measurements, the changes in water dissociation rate at the junction become slower at lower frequencies and the hydration state of the BPM junction changes over time, changing its conductivity. Since there is no real understanding of what is exactly happening at such low frequencies and how to model it, the low-frequency data were also not included in the fit.

**Figure 7:**
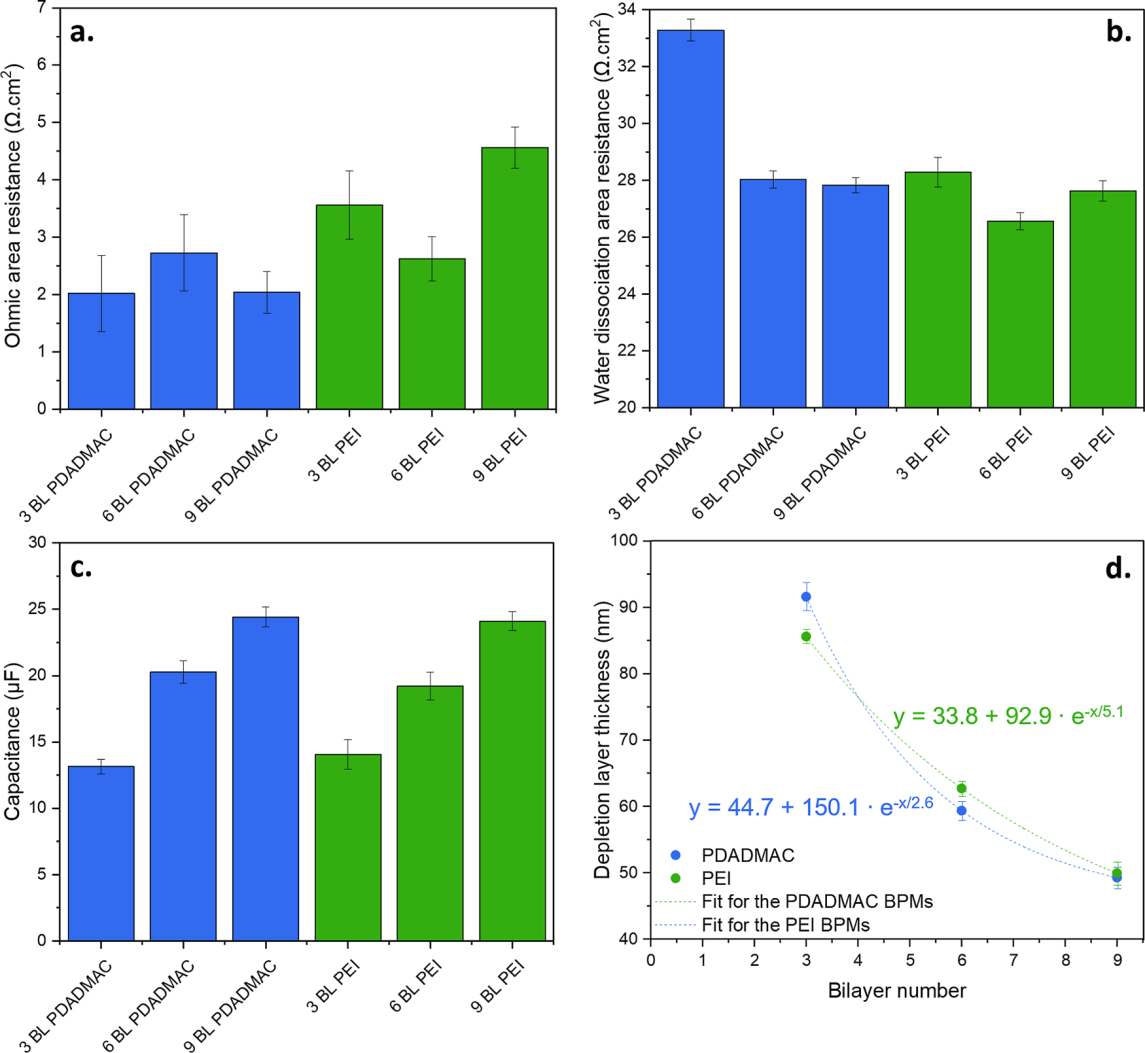
Extracted values from fitting the EIS Nyquist plots (Figure 6) with the equivalent circuit plotted in Figure 6b. (a) Ohmic area resistance of the BPMs (corrected for the solution resistance). (b) Water dissociation area resistance. (c) Capacitance of the BPM junction. (d) Depletion layer thickness extracted from the capacitance values. In all cases, the error bar represents the standard error from the Zfit of the EC-Lab software.

The data extracted from the EIS measurements after fitting are displayed in Figure 7. The ohmic area resistance (see Figure 7a) is similar for all the membranes and the deviations between samples are related to the deviations in BPM thickness. For example, the 9 BL PDADMAC BPM has the lowest ohmic area resistance but is also the thinnest membrane (70 μm thick in Figure 4), while the 9 BL PEI BPM is the thickest (76 μm in Figure 4) and has the highest ohmic area resistance. This correlation is observed since the ohmic area resistance is mainly determined by the ion transport through the AEL and CEL, which are of the same material and of similar thickness for all the BPMs.

The main differences between the BPM performances are due to the water dissociation area resistance (see Figure 7b) and the capacitance (see Figure 7c). In good agreement with Figure 5b,c, the water dissociation area resistance of the PDADMAC samples drastically decreases from 3 to 6 BL, after which it remains constant. This is attributed to more clay adsorption from 3 to 6 BL, and a maximum value being reached at 6 BL PDADMAC. For the PEI BPMs, the water dissociation area resistance data from the impedance measurements do not match the trends seen in the *J*–*V* curves. According to the EIS results, the water dissociation area resistance is the smallest for the 6 BL PEI sample, while the *J*–*V* curves suggest that the 3 BL PEI is the best-performing PEI BPM. This is because the difference between the PEI membranes is not yet significant at low current densities. Overall, the water dissociation area resistance is slightly higher than that reported for graphene oxide and PDADMAC bilayers (15 Ω.cm^2^), due to graphene oxide being a better water dissociation catalyst than K30 MMT.^16^

Interestingly, the capacitance (in Figure 7c) is rather similar for both polymers and is more dependent on the number of bilayers rather than on the used polycation. The capacitance is a measure of the amount of charges that the BPM junction can store at a given potential and is related to the depletion layer thickness according to eq 5 (assuming that the junction behaves as a parallel plate capacitor)^16^
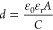
5

In this equation, ε_0_ is the vacuum electric permittivity (8.85 × 10^–12^ F/m), ε_r_ is the dielectric constant (78.4 for pure water at ambient conditions) at the junction of the BPM, *A* is the membrane area (1.735 × 10^–3^ m^2^) and *C* is the capacitance (F).^16,50^

The extracted depletion layer thicknesses (Figure 7d) decay exponentially with an increasing number of bilayers, indicating that there is very quickly a limited benefit of adding more bilayers in the BPM junction, as was concluded from the *J*–*V* curves as well. Thinner depletion layers are usually attributed to better-performing BPMs as a thinner depletion layer indicates a faster H^+^ and OH^–^ production rate at the junction as the water dissociation happens to a larger extent via the chemical reaction model than through the SWE.^16^ These results are in the same order of magnitude and are in line with those of Yan et al. for graphene oxide/PDADMAC multilayers.^16^

Interestingly, the extrapolated depletion layer thickness at 0 bilayers is higher for PDADMAC (194.8 nm) than for PEI (126.7 nm), whereas one would expect this value to be similar for both cases. Unfortunately, this value could not be determined experimentally due to the high resistance of the blank BPM (without composite multilayer junction), which creates voltages that are outside the bounds of the potentiostat. One major assumption made in the depletion layer thickness computation is that the junction area is the same as the BPM geometric area, meaning that the junction area is highly underestimated. The discrepancies between the extrapolated thicknesses at the junction could suggest that the PEI coatings are rougher than the PDADMAC coatings. This is in line with the findings of Voigt et al., who correlated lower polyelectrolyte charge density to rougher layer formation.^51^ Therefore, this could indicate that capacitance values cannot be used to determine quantitively the depletion layer thickness without an accurate area measurement, but only to give a qualitative trend within a set of similar materials.

### BPM Permselectivity

**Figure 8:**
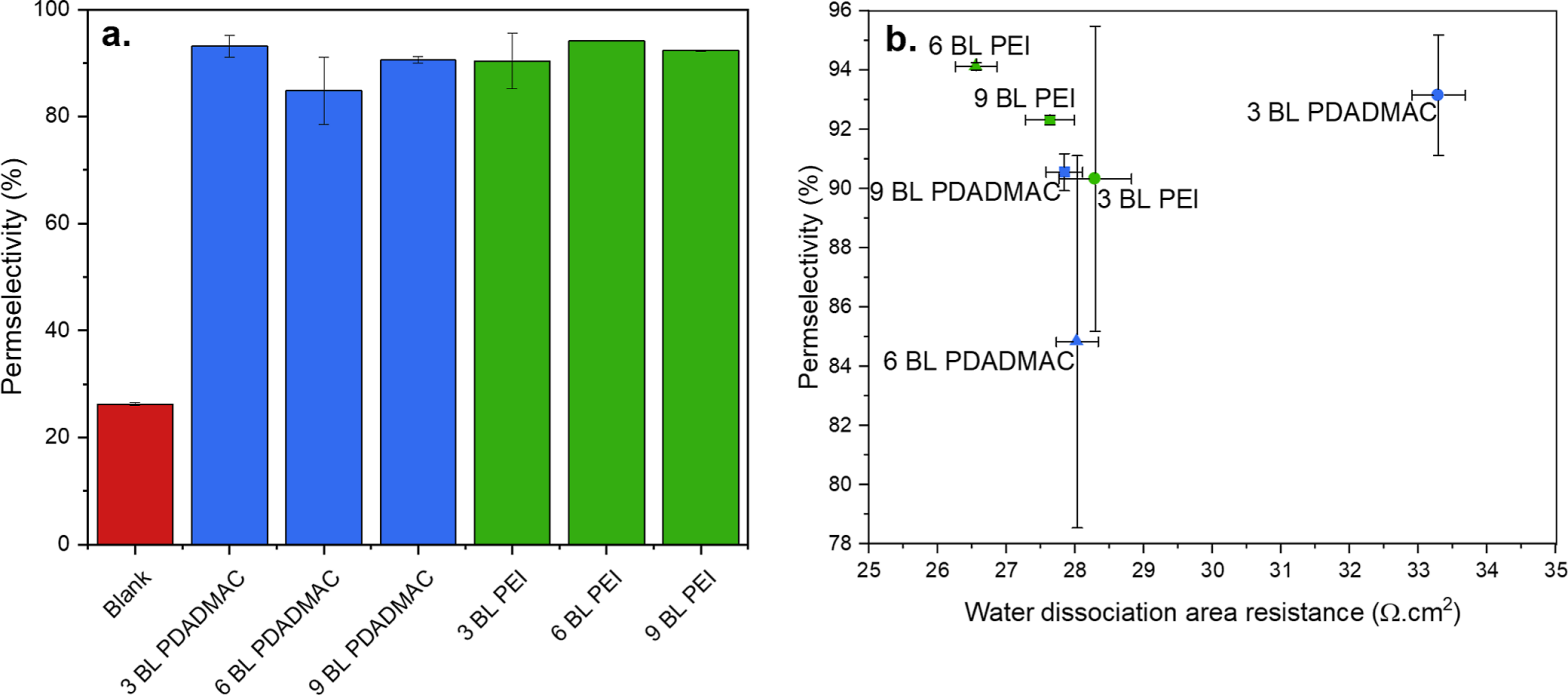
(a) Permselectivity of the made BPMs for 0.5 M HCl and 0.5 M NaOH. The blank membrane refers to the BPM made without a catalyst. (b) Permselectivity vs water dissociation area resistance (from Figure 7b) for the BPMs with composite multilayers.

The results presented show a decrease in BPM overpotential with the presence of MMT composite bilayers at the junction. However, achieving a low water dissociation resistance is of limited benefit for electrochemical processes if the BPM is not selective. Therefore, the BPMs permselectivity was measured and is reported in Figure 8.

Figure 8a shows that the addition of composite bilayers in the BPM junction greatly enhances the BPM selectivity. Even for the least selective membrane (6 BL PDADMAC), the permselectivity is improved by a factor of 3.2, and the best permselectivities are similar to those of the Fumasep FBM (95% according to the results of Al-Dhubhani et al.).^40^ The permselectivity of the BPMs with composite multilayers does not show significant differences between one and the other, indicating that the presence of three bilayers is enough to improve the selectivity. These results are rather peculiar as the CEL and AEL are typically mentioned as the source of the BPM selectivity while the junction’s function is mainly the water dissociation reaction catalysis.^13,52^ This improvement can be thanks to the ionic cross-links between the outer clay layer on the SPEEK and the adjacent FAA-3, which increases the local selectivity in the AEL next to the junction. Furthermore, the LbL coatings contain a high concentration of fixed charges, which also contributes to a higher Donnan exclusion in the BPM junction. Finally, the denser structures of the coated BLs compared to the CEL and AEL can also contribute to an increased permselectivity.

Typically, BPMs with high permselectivities and low water dissociation resistance are desirable for electrochemical processes. Figure 8b displays the permselectivity vs the water dissociation resistance of the BPMs with polycation-MMT composite multilayers. Ideally, the most suitable BPMs are in the top left corner of the graph. Within this work, the best BPM is the 6 BL PEI as it has the highest permselectivity and the lowest water dissociation area resistance. To demonstrate the advantage of using BPMs with such composite multilayers in their junction, the 6 BL PEI and 6 BL PDADMAC BPMs are tested for their application in acid and base production via BPM electrodialysis.

### Acid and Base Generation via BPM Electrodialysis with BPMs Containing Composite Multilayers-Coated Junctions

**Figure 9:**
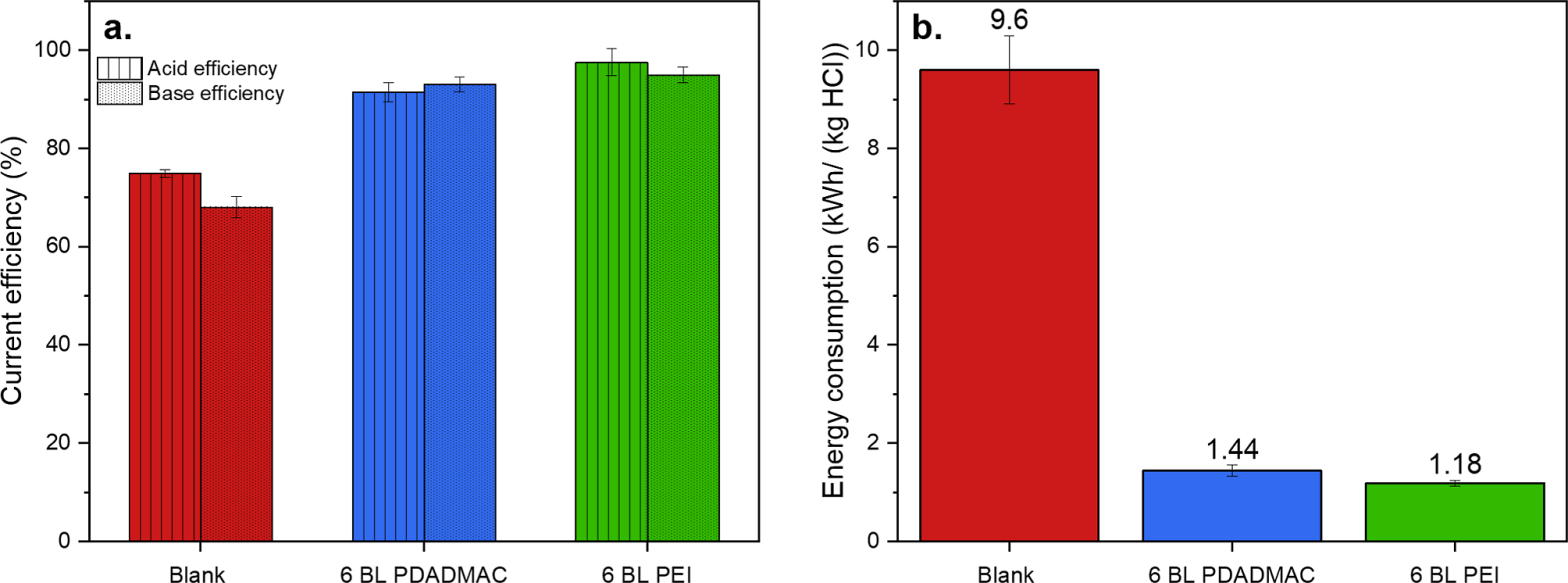
(a) Current efficiency and energy consumption of the blank, 6 BL PDADMAC and 6 BL PEI BPMs and (b) their energy consumption for the production of 1 kg HCl.

The current efficiency (a) and energy consumption (b) for the production of acid and base are shown in Figure 9 for the blank, 6 BL PDADMAC and 6 BL PEI BPMs.

Figure 9a shows that the current efficiency for acid production increases following the order: blank < 6 BL PDADMAC < 6 BL PEI. This order follows the expected outcome given the measured permselectivites reported in Figure 8a. The higher the BPM permselectivity, the lower the unwanted acid and base crossover through the BPM, and therefore the higher the current efficiency. As a result of the higher current efficiency, as well as the lower overpotential for the water dissociation reaction, the energy consumption for the production of 1 kg HCl is at least 6.7 times lower when using the modified BPMs rather than using the blank BPM that does not contain any catalyst.

## Conclusion

Bipolar membranes with a junction containing 3, 6, and 9 MMT clay composite bilayers as a catalyst for the water dissociation reaction were fabricated with a strong polyelectrolyte (PDADMAC) or a weak polyelectrolyte (PEI). The strong PDADMAC polyelectrolyte leads to the formation of stable bilayers and adsorption of the MMT clay, while the weak PEI polyelectrolyte leads to less clay adsorption and unstable layers at a higher number of BLs, as desorption of the clay was noticed when going from 6 to 9 BL PEI. In all cases, the presence of the composite multilayers leads to a 26-fold decrease in the water dissociation resistance compared to a BPM fabricated without a catalyst. This is thanks to the addition of the MMT clay that catalyzes the water dissociation reaction thanks to its surface silanol groups. Interestingly, while the water dissociation and ohmic resistances are relatively similar for all the MMT clay-containing BPMs, it is found that the capacitance increases drastically with the number of bilayers as the junction depletion layer decreases exponentially with the number of bilayers. This is due to the increase in clay content, which changes the water dissociation mechanism from the second Wien effect to the chemical reaction model. Surprisingly, the permselectivity of the BPMs is also tripled by the addition of the multilayers in the BPM junction, which is attributed to the denser LbL structures and their higher charge density. Moreover, the enhanced BPM performance leads to at least a 7-fold decrease in the energy needed for the production of 1 kg of hydrochloric acid for the 6 BL-coated BPMs. Therefore, the improvements in the BPM water dissociation kinetics and permselectivity by the addition of clay-composite multilayer catalysts increases the energy efficiency of electrochemical processes significantly.

## Supporting Information Available

Optical pictures of the six-compartment and two-compartment setups, and full and zoomed-in XPS spectra (PDF)
