# Supplementary material for: Using Layer-by-layer Assembled Clay Composite Junctions to Enhance the Water Dissociation in Bipolar Membranes
Source: Langmuir. 2024 Nov 13;40(47):24795–807. doi: 10.1021/acs.langmuir.4c02514 (PMC11603776; doi:10.1021/acs.langmuir.4c02514)
Supplement: Supplementary file 1 — la4c02514_si_001.pdf [file la4c02514_si_001.pdf]

USING LAYER-BY-LAYER ASSEMBLED CLAY COMPOSITE JUNCTIONS TO ENHANCE THE WATER DISSOCIATION IN BIPOLAR MEMBRANES

---- SUPPORTING INFORMATION ----

*AUTHOR NAMES: Nadia Boulif, Menno Houben, Zandrie Borneman, Kitty Nijmeijer**

**Corresponding author: d.c.nijmeijer@tue.nl*

AUTHORS ADDRESS: Membrane Materials and Processes, Department of Chemical Engineering and Chemistry, Eindhoven University of Technology (P.O. Box 513, 5600, MB, Eindhoven, The Netherlands)

KEYWORDS: Bipolar membrane, Montmorillonite clay, Layer-by-layer, Composite multilayers, Water dissociation reaction.

Supporting information 1. Optical pictures of experimental setups

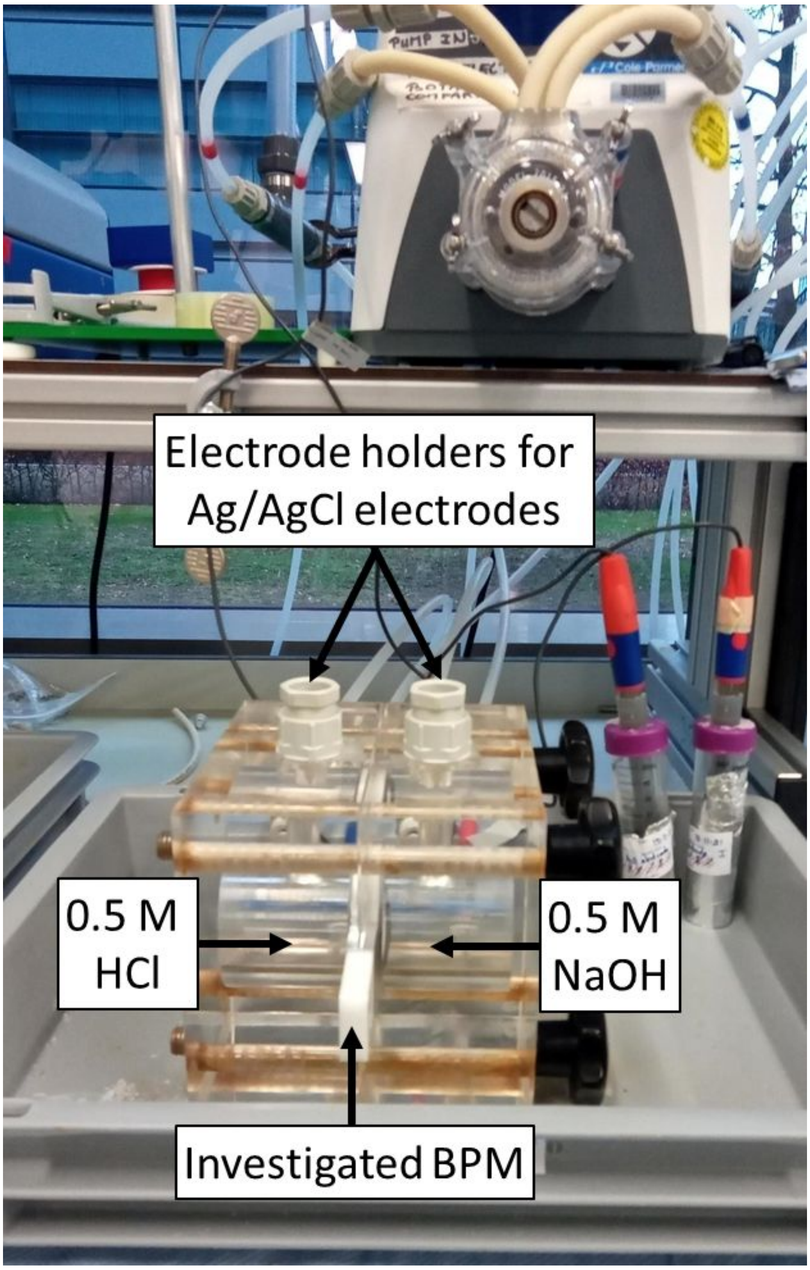

Figure S.I.1.1. *Optical picture of the permselectivity setup. The investigated BPM is placed in between an acid (0.5 M HCl) and a base (0.5 M NaOH) solution which is recirculated throughout the measurement, and the open cell voltage is monitored over time.*

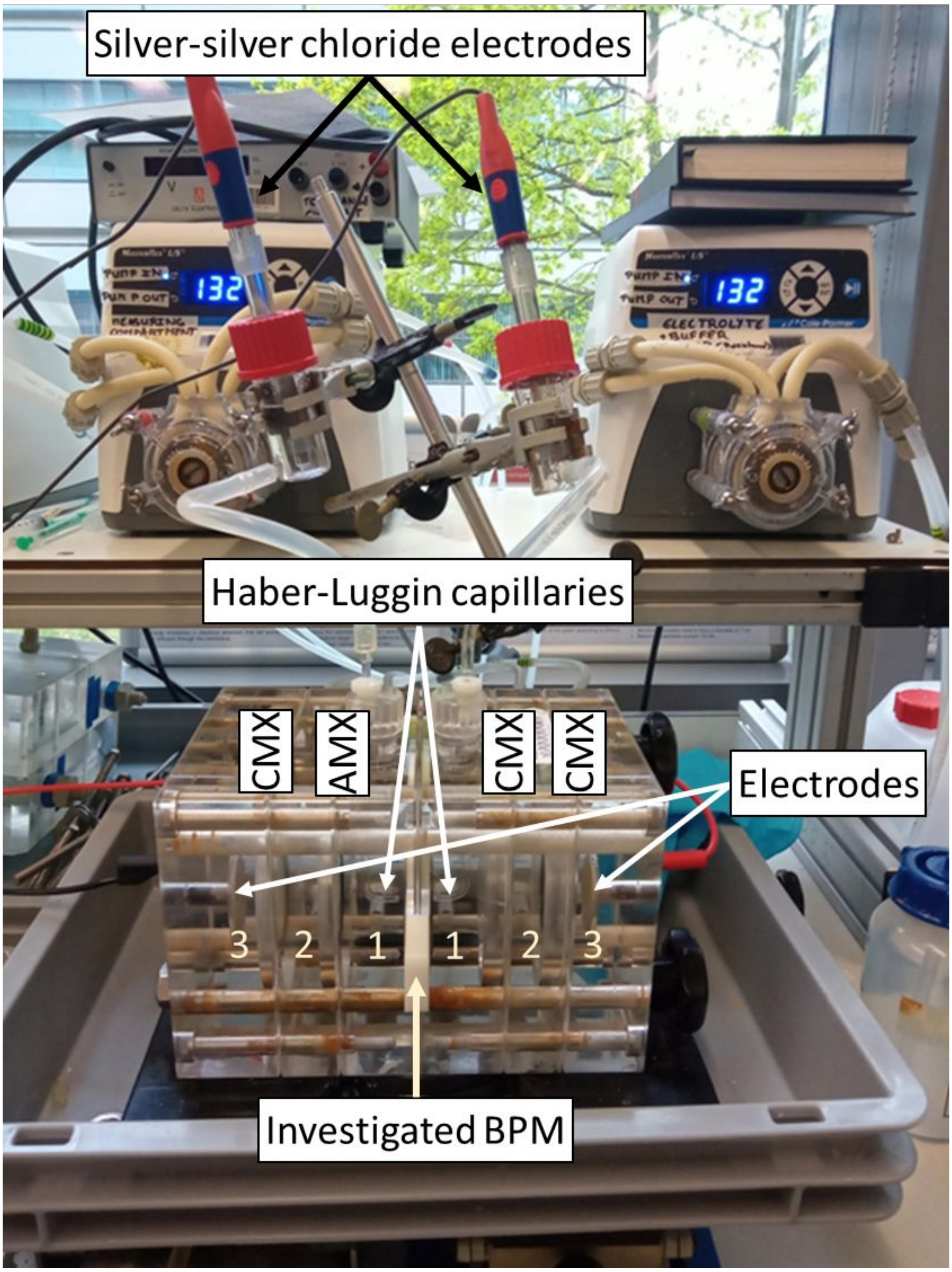

Figure S.I.1.2. *Optical picture of the six compartment cell. The numbers 1,2, and 3 correspond to the measuring (0.5 M NaCl), buffer (0.5 M NaCl) and electrolyte (0.5 M* Na_2SO_4*) compartments, respectively, which are connected to the same flasks. CMX and AMX are referring to the commercial Neosepta CMX-fg and Neosepta AMX-fg membranes that are used to separate the solution compartments. The electrodes are platinized titanium.*

Supporting information 2. XPS spectra

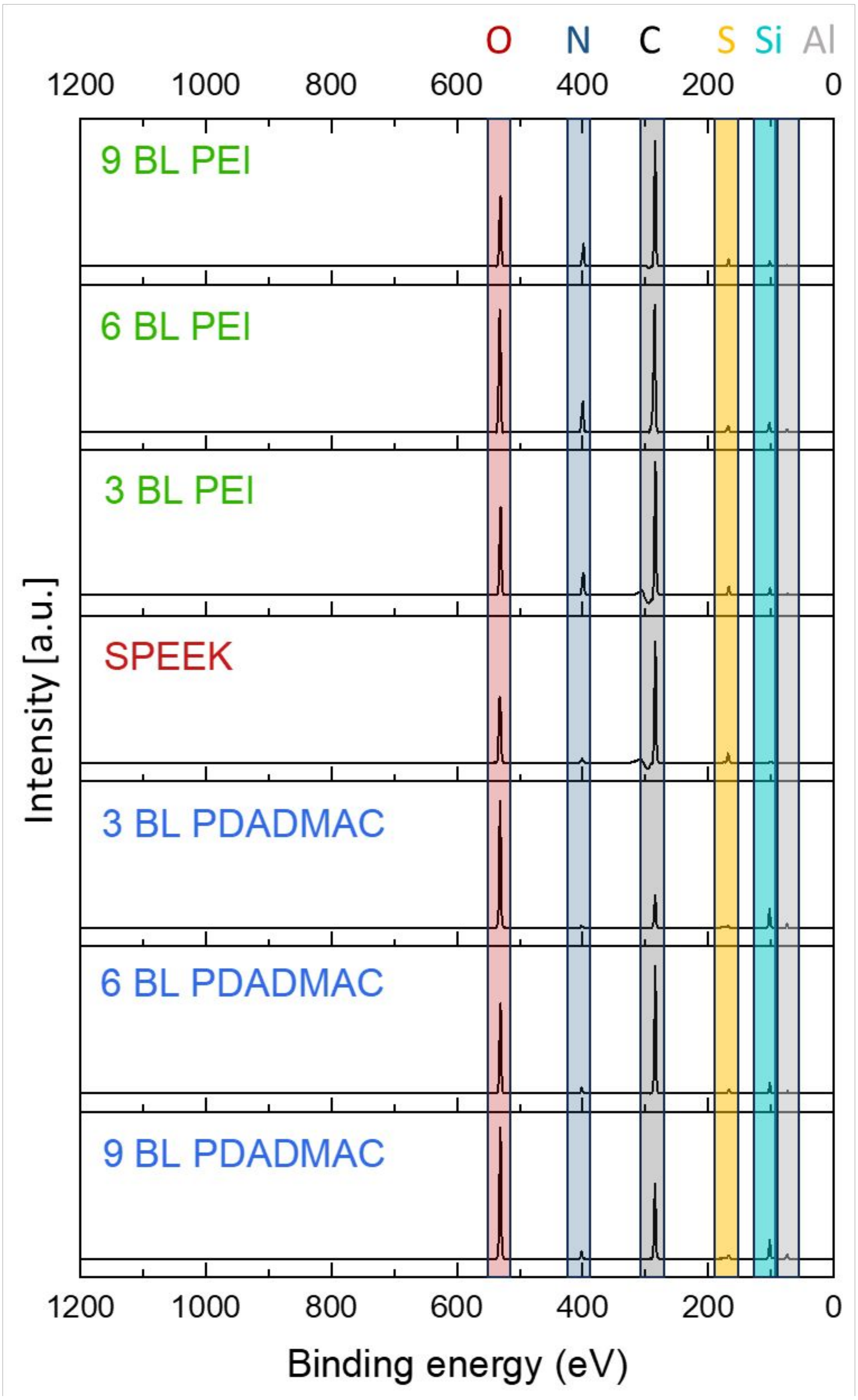

Figure S.I.2.1*. XPS spectra of the SPEEK fibers coated with layer-by-layer with K30 MMT and a polycation. The amount of bilayers and the polycation type is indicated for each spectra.*

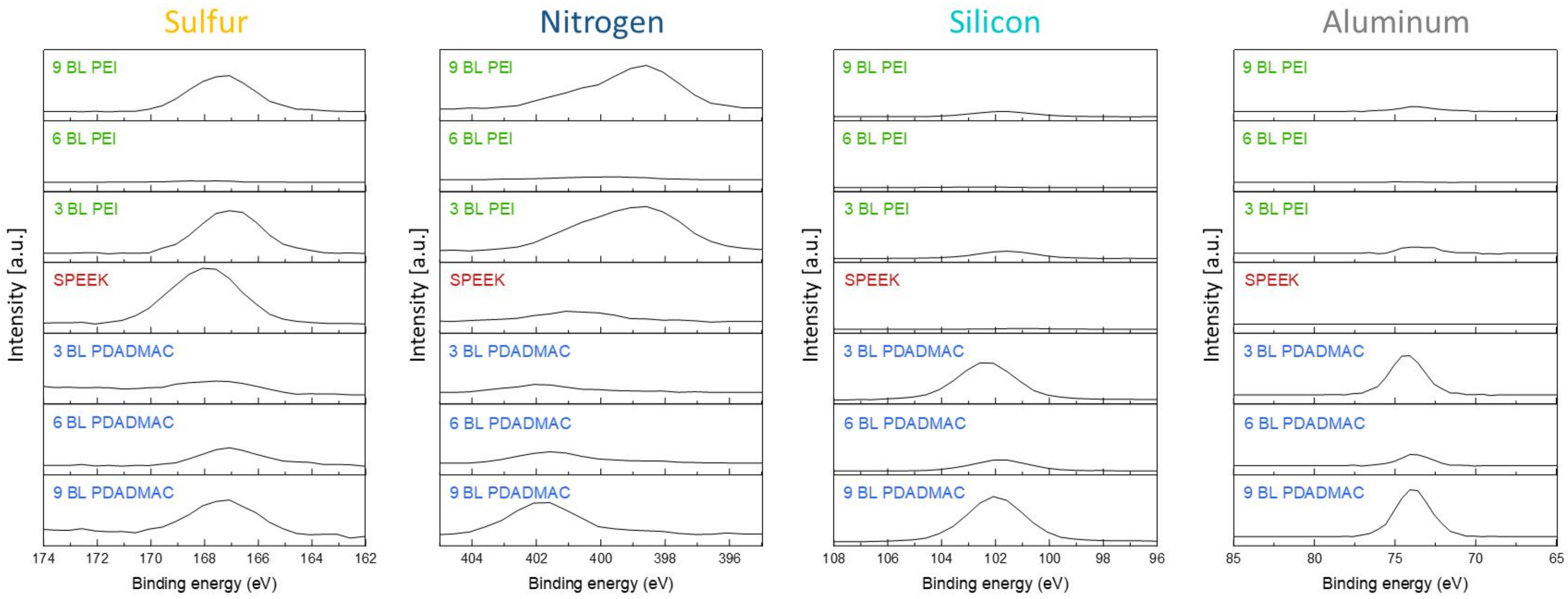

Figure S.I.2.2. *Zoom into the region of the XPS spectra (shown in Figure S.I.2.1) used to determined the nitrogen, silico and aluminum ratio to sulfur. The zoom is different between the different element but the y-axis is similar between the different samples.*
